# Transcutaneous Cervical Vagus Nerve Stimulation Induces Changes in the Electroencephalogram and Heart Rate Variability of Healthy Dogs, a Pilot Study

**DOI:** 10.3389/fvets.2022.878962

**Published:** 2022-06-13

**Authors:** Gibrann Castillo, Luis Gaitero, Sonja Fonfara, Christopher J. Czura, Gabrielle Monteith, Fiona James

**Affiliations:** ^1^Department of Clinical Studies, Ontario Veterinary College, University of Guelph, Guelph, ON, Canada; ^2^Convergent Medical Technologies, Inc., Oyster Bay, NY, United States

**Keywords:** vagus nerve stimulation, transcutaneous cervical vagus nerve stimulation, frequency band analysis, power spectral analysis, heart rate variability, SDNN index

## Abstract

Transcutaneous cervical vagus nerve stimulation (tcVNS) has been used to treat epilepsy in people and dogs. Objective electroencephalographic (EEG) and heart rate variability (HRV) data associated with tcVNS have been reported in people. The question remained whether EEG and electrocardiography (ECG) would detect changes in brain activity and HRV, respectively, after tcVNS in dogs. Simultaneous EEG and Holter recordings, from 6 client-owned healthy dogs were compared for differences pre- and post- tcVNS in frequency band power analysis (EEG) and HRV. The feasibility and tolerance of the patients to the tcVNS were also noted. In a general linear mixed model, the average power per channel per frequency band was found to be significantly different pre- and post-stimulation in the theta (*p* = 0.02) and alpha bands (*p* = 0.04). The pooled power spectral analysis detected a significant decrease in the alpha (*p* < 0.01), theta (*p* = 0.01) and beta (*p* = 0.035) frequencies post-stimulation. No significant interaction was observed between dog, attitude, and stimulation in the multivariate model, neither within the same dog nor between individuals. There was a significant increase in the HRV measured by the standard deviation of the inter-beat (SDNN) index (*p* < 0.01) and a decrease in mean heart rate (*p* < 0.01) after tcVNS. The tcVNS was found to be well-tolerated. The results of this pilot study suggest that EEG and ECG can detect changes in brain activity and HRV associated with tcVNS in healthy dogs. Larger randomized controlled studies are required to confirm the results of this study and to assess tcVNS potential therapeutic value.

## Introduction

Vagus nerve stimulation (VNS) is a non-pharmacologic treatment option in human patients with epilepsy (1–3). This technique consists of providing controlled electrical stimulation of the vagus nerve, either through a device surgically implanted on the left cervical vagus nerve or through a non-invasive transcutaneous unit (2, 4). Transcutaneous cervical vagus nerve stimulation (tcVNS) has been reported as an adjunctive treatment in drug resistant epilepsy and other diseases like depression, migraines or pain (5–8).

In veterinary medicine, the estimated prevalence of dogs considered resistant to conventional anti-seizure therapy is ~25% (9). This high prevalence drives the investigation of non-pharmaceutical therapies like VNS. A surgically implanted VNS device has been studied in dogs (10). Despite the favorable response observed in some dogs, the cost, unreliable functionality and short- and long-term complications make a surgically implanted device inaccessible for many drug-resistant patients (10, 11). The tcVNS apparatus in dogs with drug resistant idiopathic epilepsy is reported to be well-tolerated; although no significant effect on the overall reported seizure frequency was found (12). Objective measures of tcVNS effects on vagus nerve activity remain to be described.

The vagus nerve has an important role in homeostasis functions. Due to its mixed composition of afferent sensory and efferent motor and parasympathetic axons, stimulation of the vagus nerve results in a plethora of different physiologic changes, with those in the brain and heart of particular interest for this study (13–15). The proposed mechanism of action of VNS on the brain is a direct increase in the release of norepinephrine from the nucleus of the solitary tract to the projections sent to the locus coeruleus and, indirectly, increasing the serotonin release by the dorsal raphe nucleus (16, 17). There is evidence that VNS results in an increase in the norepinephrine concentration in healthy Beagles, further suggesting that an increase in this monoamine could play an important role in the mechanism of action of VNS (18). The changes in the concentration of these neurotransmitters presumably influence the synaptic plasticity of the neurons, resulting in an anti-seizure effect (16, 17, 19, 20). Another change that has been identified in healthy dogs undergoing VNS is a decrease in the perfusion of the frontal lobe, which has been hypothesized could contribute to the mechanism of action of VNS (21). While it is safe to stimulate either side of the vagus nerve, stimulation of the right branch and sinoatrial node is more likely to result in bradycardia and asystole (11, 22). Dogs, as opposed to humans, have a more caudal branching of cardiac motor fibers innervating the atrioventricular node on the left side of the heart, therefore stimulation of the left side of the vagus nerve could still result in bradycardia and asystole (11, 23). Notably these cardiovascular complications have been observed with surgically implanted but not with transcutaneous devices in dogs (12).

The effect of VNS and tcVNS on the cardiovascular system has been studied in people and animals (24–29) mainly by means of heart rate variability (HRV). This is defined as the variation in the interval between successive R waves (NN or R-R intervals) in the cardiac cycle, and is considered to be an indicator of the autonomic nervous system activity (30–33). The standard deviation of the inter-beat (NN or R-R) interval in a sinus rhythm is termed SDNN, and the mean of the standard deviations of the normal inter-beat intervals in a given period of time is called the SDNN index (34). This is considered the most reliable way to assess the autonomic function of the heart (34). The decrease in heart rate and the increase in HRV observed with VNS is secondary to the release of acetylcholine at the sinoatrial node (35).

In people, tcVNS has been documented to result in identifiable changes in brain activity. Electrical brain activity is registered by electroencephalography (EEG), recorded via electrodes and then transferred to a computer system (36, 37). Brain activity changes in people after tcVNS include a decrease in abnormal EEG patterns in patients with epileptic seizures, attenuation of the alpha rhythm, and an overall decrease in the theta and alpha frequency bands power (5, 38, 39). Power is a form of quantitative EEG analysis that divides the signal into bands based on their frequencies; this is known as frequency band analysis (40–42). The different frequency bands are historically designated as beta (13–30 Hz), alpha (8–13 Hz), theta (4–8 Hz) and delta (1–4 Hz) (43). Spectral analysis of these frequency bands calculates the power, the relative strength of the frequencies in a signal within a determined time period of the EEG recording, known as an epoch (41, 44, 45). This quantitative analysis is useful in detecting alterations to normal brain activity associated with changes in the resting state or in a disease process.

Based on this research into the effects of tcVNS on the brain and heart in people, both EEG and HRV have been shown to provide objective measures. Objective measurement of these outcomes would support the determination of optimal tcVNS dose and response for dogs. Before establishing tcVNS as a potential supplementary treatment for drug-resistant epilepsy in dogs, it is important to determine if tcVNS results in objective physiologic changes and, if it does so, what are the expected physiologic changes to be registered.

The objectives of this pilot study were: (1) to determine if tcVNS could induce changes in frequency band spectral analysis as assessed by EEG and changes in the HRV as assessed by Holter monitoring, and (2) to assess the tolerability of the tcVNS device in dogs.

## Materials and Methods

### Animals

Six healthy community owned dogs were recruited for this prospective pilot study. As the first study of its kind, the effect size of this intervention has not been established from previous pilot studies nor has the minimum difference considered to be significant been determined. The sample size was proposed based on previous studies that successfully assessed the effect of drugs or the use of different electrodes on EEG recordings (7, 11, 46). The dogs were enrolled from the teaching hospital staff, university staff and referring veterinarian community. Dogs included in the study had to be older than 1 year but under 10 years of age and healthy based on a complete physical and neurological examination performed by one of the authors (GC). The patients were placed in a quiet room under the supervision of GC for the duration of the recording period. The Institutional Animal Care and Use Committee of the University of Guelph approved the animal use protocol (# 4265) for this study and the protocol followed the Canadian Council on Animal Care guidelines. Written informed consent was obtained from the owners for the participation of their animals in the study.

### Holter Monitor

The Holter monitoring was performed using a digital Lifecard CF 3 channel Holter recorder (Spacelabs Healthcare, Snoqualmie, WA) that was fitted in a snug jacket after securing the leads to the chest with sticky ECG pads and adhesive medical tape right after the physical and neurological examinations were completed. The ECG recording was started immediately after the Holter instrumentation was completed but data was not collected for analysis until the remaining study instrumentation was complete.

After the Holter was placed, a 10 cm in length x 4 cm in width area of fur was clipped over the left jugular groove over the carotid pulse where the tcVNS therapy was to be delivered.

### EEG

**Figure 1:**
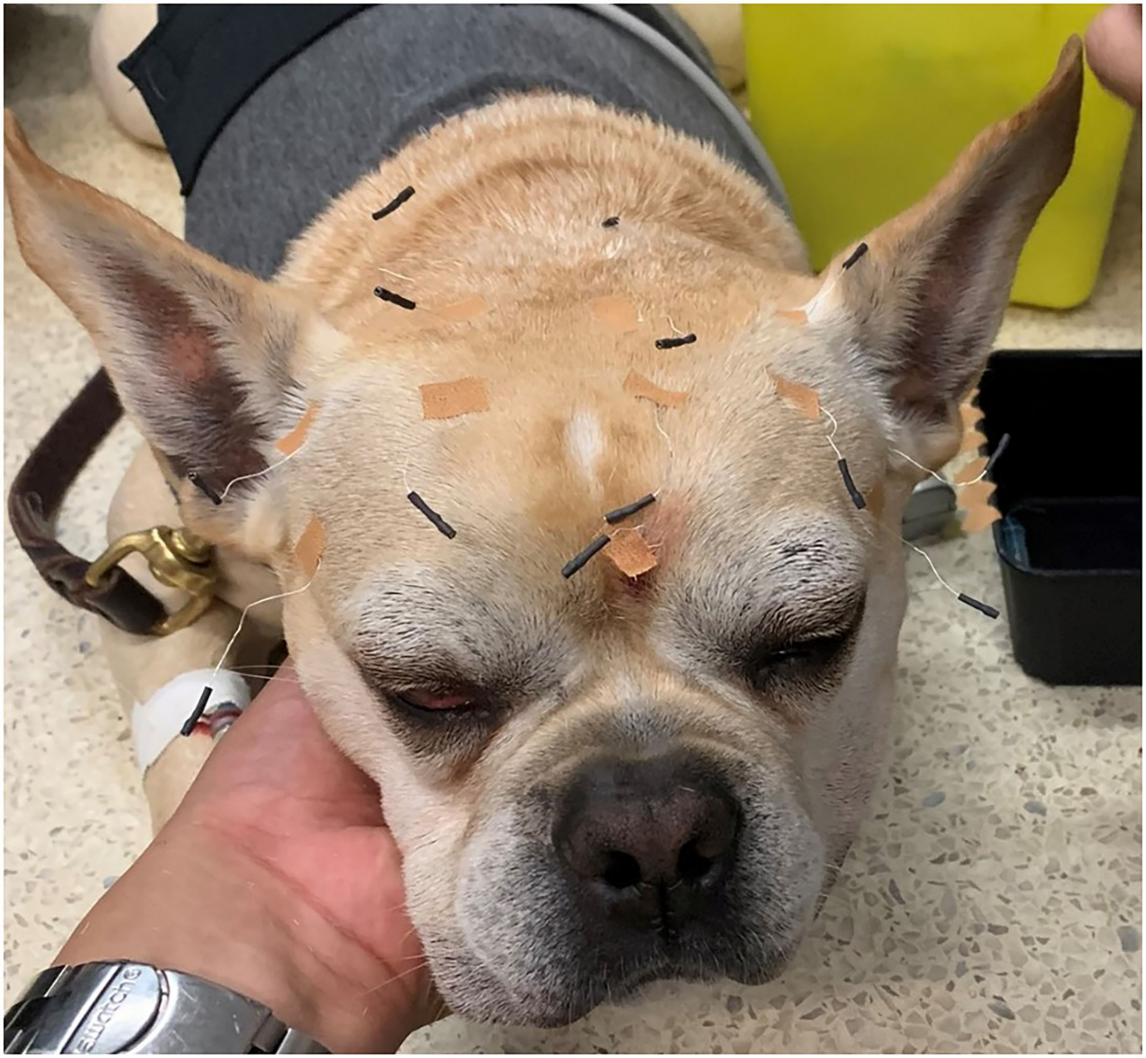
Electrode placing and fixation with sticky tape on a dog sedated with propofol.

**Figure 2:**
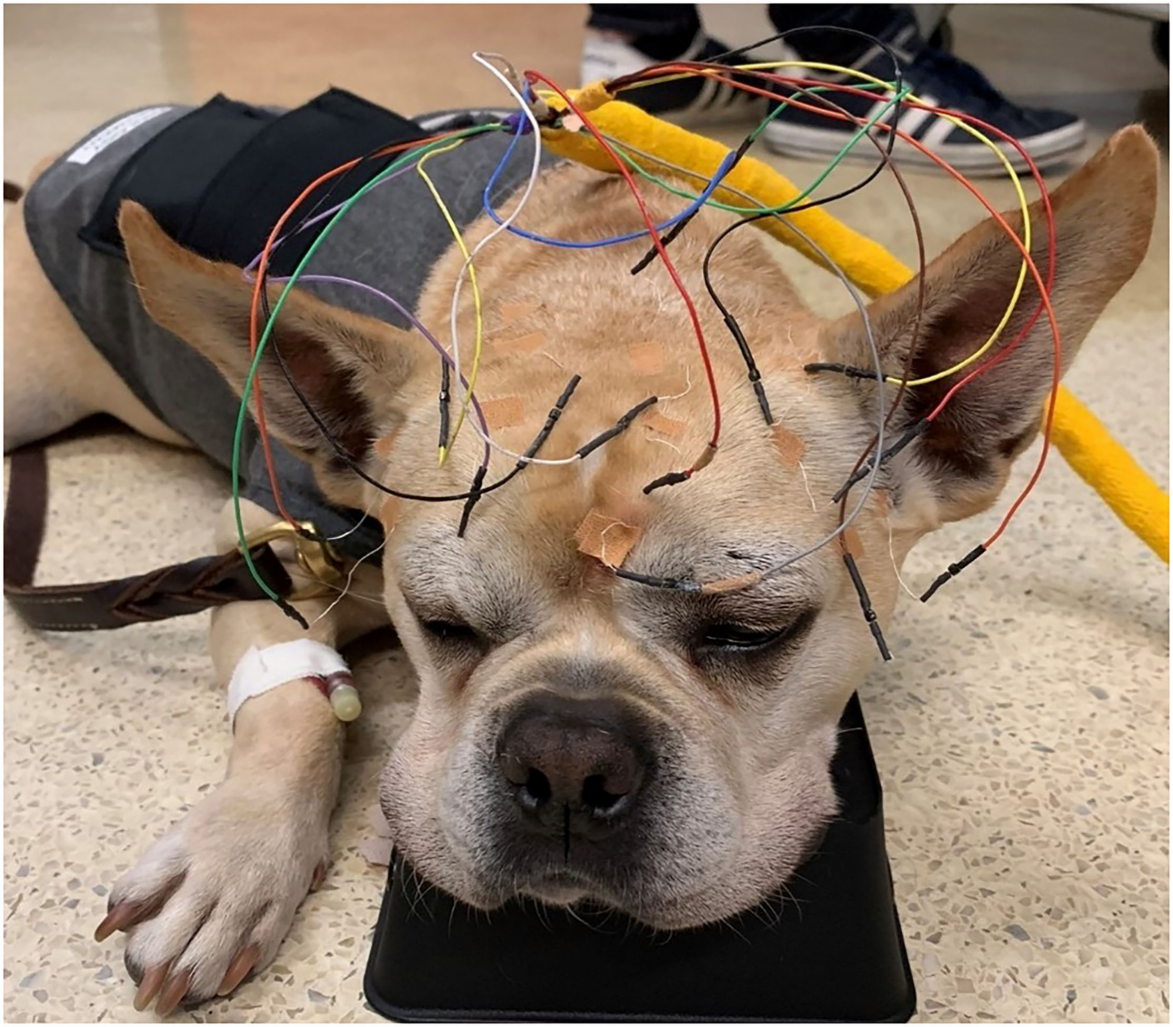
Leads attached to the electrodes prior to the placement of the head bandage.

**Figure 3:**
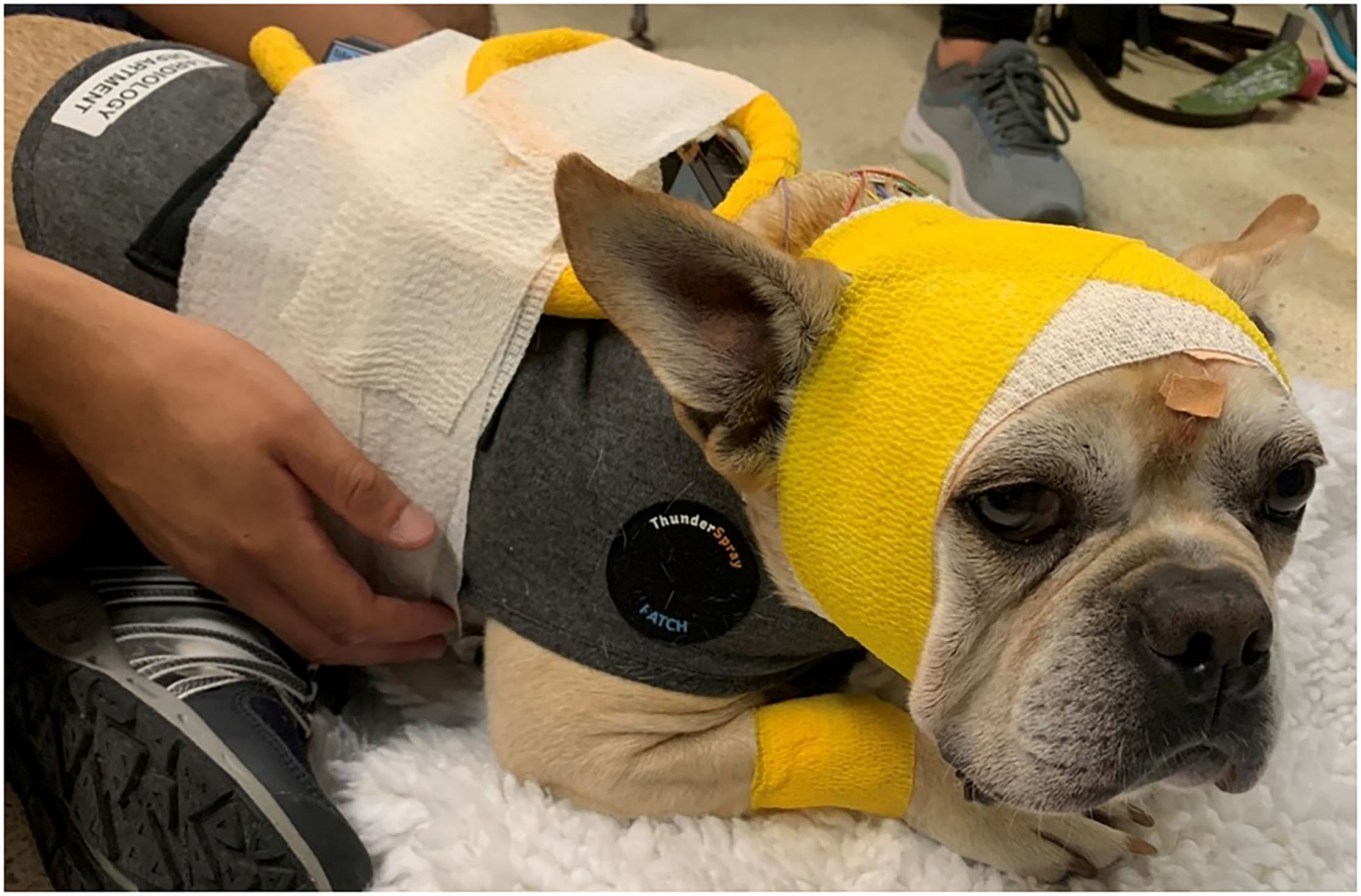
Head bandage placed after the dog recovered from the sedation with propofol. A snug-fitting jacket has been placed to secure the Holter monitor and the TrackIt device.

After the Holter placement, the EEG instrumentation was performed with subdermal wire electrodes loaded in 25-gauge needles. Electrode placement was the same as described in a previous study (47). EEG instrumentation was first attempted without sedation, however for dogs whose temperament prevented the placement of the electrodes, a 22-gauge catheter was placed in a peripheral vein (cephalic or saphenous vein). Following catheter placement, a 6 mg/kg bolus of propofol was slowly administered IV to the level required to achieve calm relaxation while maintaining jaw tone, palpebral reflexes, and spontaneous breathing. Then 2 mg/kg boluses of propofol were given as needed to complete instrumentation. Femoral and pedal pulses along with the respiratory rate were constantly monitored during the 20–40 min of the EEG instrumentation phase. The electrodes were kept in place by using sticky bandage and both electrodes and leads were safely secured with loosely placed elastic sticky bandage around the dog's head and neck. Finally, the wireless transmitter TrackIt MK3 EEG recorder with video (Lifelines Neurodiagnostics Systems, Troy, IL, USA) was placed in the same snug jacket of the dogs (Figures 1–3). The EEG synchronized video camera recording was started at the same time as the instrumentation and the angle of the camera was kept in a position that allowed the greatest possible visualization of the dog. At the end of instrumentation, impedance was checked, and recording was started only when all the electrodes showed an electrical impedance under 30 kOhms (48). After the sedation for the instrumentation wore off, an e-collar was placed on one of the dogs for the rest of the recording to avoid electrode removal.

### Recording and Stimulation

Once that both Holter and EEG devices were safely placed and dogs were completely awake from the sedation and able to stand up on their own, 1-h of pre-stimulation (basal) ECG and EEG were recorded followed by 120 s of stimulation and finally by 1-h of ECG and EEG recording post-stimulation. Even though the EEG and Holter devices had started recording right after placement, the 1-h pre-stimulation period used for analysis was not counted until after the dogs had recovered from sedation as described above. This was to avoid any potential changes in the EEG and ECG induced by manipulation of the dogs as they were being instrumented (e.g., clipping the fur, placing IV catheter, and fitting the jacket). Thereafter, the dogs were supervised but undisturbed in the room except for the 120-s period of stimulation. During the entire recording session, the dogs were allowed to explore the room and were provided with a bowl of water and comfortable bedding to lie down if they preferred to.

For the stimulation period, contact gel was placed on the previously clipped spot on the neck and the tcVNS device (gammaCore-VET; ElectroCore, LLC, New Jersey, United States) was placed making sure that both electrodes were in direct contact with the skin by applying light pressure. The gammaCore instrument is programmed by the manufacturer to deliver 120 s of stimulation after which the device automatically turns off. The device delivers an electrical signal consisting of a 5-kHz sine wave burst lasting for 1 ms (5 sine waves, each lasting 200 ms), with such bursts repeated once every 40 ms (25 Hz) for 2 min per stimulation, as described previously (49). During the first 30 s, using the graded thumbwheel on the side of the tcVNS (graded from 1 to 5, 5 being the highest stimulation intensity), the dog's highest tolerable dose was identified. The device was started at 1 and the intensity slowly increased every 5 s. If the dog showed any signs of discomfort (yelp, growl, or movement of the head away from the device) or if marked muscle fasciculations were observed at the site of the stimulation, the intensity was decreased by 0.5 and the stimulation level was recorded as the highest tolerable dose. The reaction of the dogs to the stimulation was recorded with a video camera pointing toward the head of the dogs for the purpose of capturing any behavioral or physiological reaction that could indicate pain or discomfort from the stimulation. The remaining 90 s of the stimulation was delivered at the highest tolerable dose.

**Figure 4:**
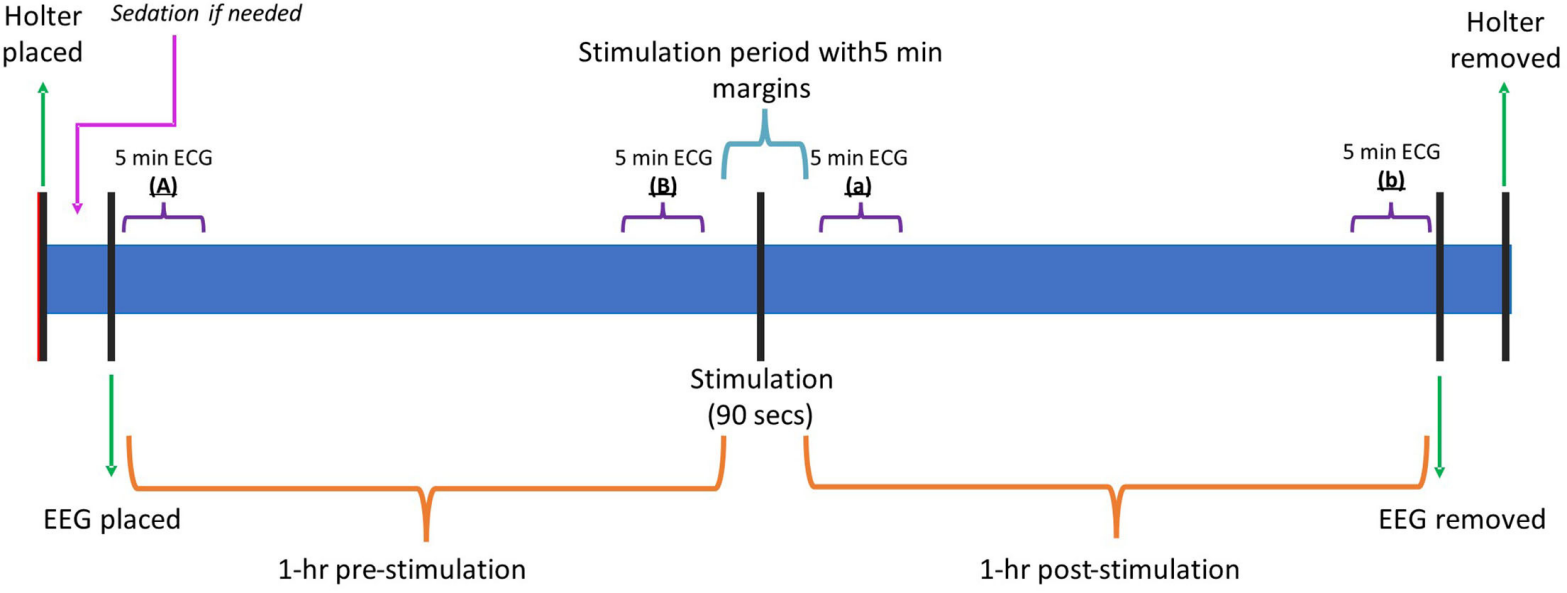
Timeline graphic summarizing the different events and key points during the recording. Min, minutes; Hr, hour; ECG, electrocardiogram; EEG, electroencephalogram.

After the remaining 90 s of stimulation were completed, another at least 1-h period of ECG and EEG were recorded in the same room under the same conditions as the first hour of recording. Figure 4 shows the timeline of events during the recording period and the data obtained.

### Frequency Band and Power Spectra Analysis

To account as much as possible for the influence of the attitude and activity of the individual dogs during the recordings, the EEG video was reviewed to pair pre- and post-stimulation segments where the patient was considered “calm” or “active” depending on their behavior. If the dog was lying down or sitting comfortably and not engaged in any activity, the segment was categorized as “calm”. A segment was labeled as “active” if the dog was standing or exploring the room. A referential montage was used to retrieve the data using 7 different channels (F3, F4, C3, C4, Cz, Fz and Pz).

**Figure 5:**
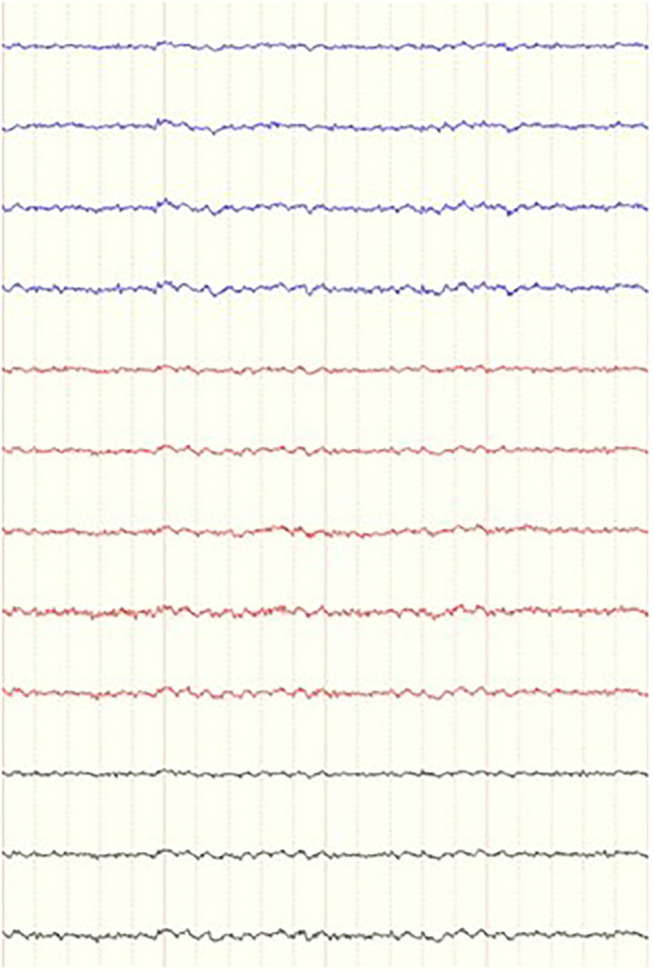
Example of a 4-s epoch of a dog in a during a period of “calm” behavior as it was presented to the blinded investigator. Referential montage, low frequency filter 0.16 ms, high frequency filter 70 Hz, notch filter 60 Hz and sensitivity of 7 uV. Ms, milliseconds; Hz, Hertz.

For each dog, several 4-second-long epochs were manually selected from the raw EEG data pre- and post- stimulation. One of the researchers (FJ), who was blinded to the signalment of the patients, the time and sequence of the recordings, and behavior, selected 9–12 epochs pre- and post- stimulation that were as artifact-free as possible (Figure 5). The 4-s epochs were then analyzed in 2 ways. Firstly, for each individual channel in each selected epoch, the average power and mean frequency values for the different frequency bands were calculated, however the algorithm in this tool (FDA Tables tool) used overlapping frequency bin borders (i.e., delta from 1 to 4 Hz, theta from 4 to 8 Hz). Secondly, for the pooled channels for each epoch, power spectral analysis of the different frequency bands, median frequency (F50) and spectral edge frequency (F95) were calculated via fast Fourier Transformation (FFT tool) with more precise frequency band borders set in the algorithm (e.g., theta from 4.1 to 8.0 Hz). Then, channel frequency and power, pooled power, F50 and F95 were compared between pre- and post-stimulation for each individual dog in both calm and active epochs. All the EEG analysis was performed using specific software (Insight II, Persyst Development Corporation, Prescott, AZ) applying a Hamming window with 256 points per window and 50% overlap in between windows.

### HRV Analysis

The data collected with the Holter monitor was analyzed with a specific software (Pathfinder SL 1.9.2 11104 version). An approximately 1-h section of the recording was selected before and after stimulation leaving a 5-min margin before and after the stimulation to account for any potential clock-time discrepancies between the Holter monitor and the registered time of stimulation. Once the 1-h long pre- and post- stimulation segments were selected, the average SDNN index and the mean heart rate values were collected from these segments. Additionally, the averaged mean heart rate of the 5-min margin before and after stimulation (for a total of 10-min-long segment) was registered.

To check whether any changes obtained when comparing the HRV and SDNN between dogs and before/after the stimulation were simply due to the dogs' habituation to the room causing a general decrease in the heart rate over the entire 2-h study, the mean heart rate and maximum heart rate for the first 5-min after the start of the EEG recording (“A”) were compared against the last 5-min prior to the stimulation (“B”). Additionally, the first 5-min of the post-stimulation period (“a”), and the 5-min prior to the end of the recording (“b”) were compared. Figure 4 shows the different 5-min intervals selected.

### Statistical Analysis

To determine the effect of VNS a general linear mixed model was used for both the individual and pooled channels' quantitative variables listed above. Fixed effects of channel, time, and attitude as well as their interactions were included in the model. Individual dog, dog plus stimulation, and dog plus stimulation plus attitude were included as random effects. When interaction terms were not significant, the models were simplified, and *p*-values were reported for the main effects. Data was checked for normality with a Shapiro Wilk test and examination of the residuals. Data was log transformed to meet the assumptions of normality. Similarly, a general linear model was run for the HRV analysis. Significance was set at *p* < 0.05.

For the 5-min segment comparisons, an ANOVA for repeated measures accounting for the correlation of structure of measures made within animals was used to test for differences in heart rate over time. Residuals were checked for normality to confirm the data met the assumptions of normality and the data was normally distributed.

## Results

The signalment of the dogs included in this study, the highest tolerable tcVNS dose and the total mg/kg of propofol used for sedation are summarized in Table 1.

**Table 1:** Signalment, stimulation level and amount of sedation used in the 6 dogs.

**Dog**	**Breed**	**Age (years)**	**Sex**	**Weight (kg)**	**Highest tolerable dose of tcVNS (1–5)**	**Propofol (mg/kg)**
1	Mix breed	2	MC	44	2	8
2	French bulldog	7	FS	13	4	10
4	Mix breed	2	FS	26.5	4	8
5	Border Collie	8	FS	19	4	None
6	Mix breed	1	FS	20	4.5	9
10	Mix breed	6	FS	22.2	3	None

*MC, male castrated; FS, female spayed; tcVNS, transcutaneous vagus nerve stimulation*.

### EEG Power and Mean Frequency Analysis per Channel

**Figure 6:**
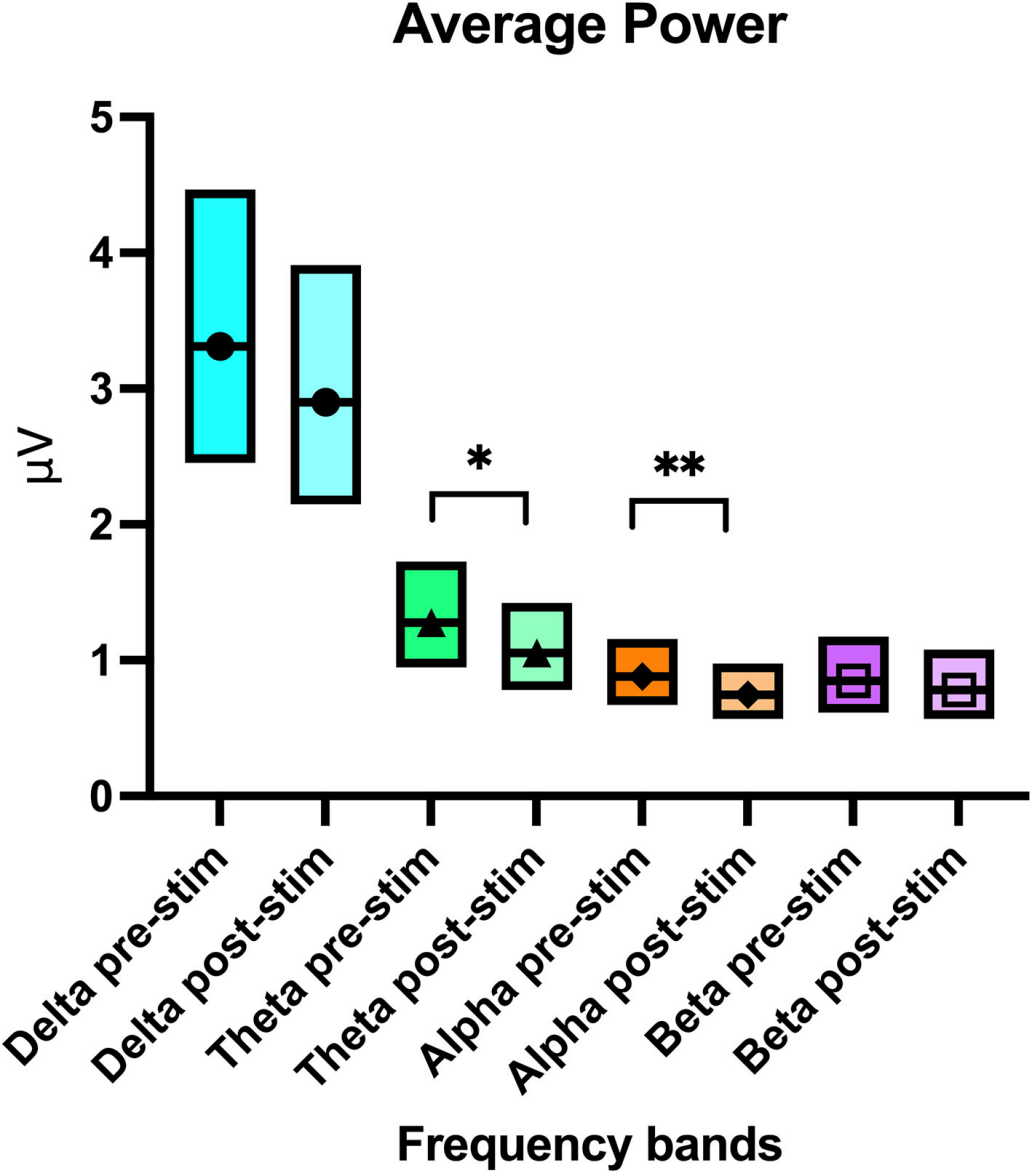
Boxplot graph showing the average power per channel per frequency band with the upper and lower confidence intervals before and after stimulation. The line crossing the boxes indicate the median value obtained in that frequency band. An * denotes statistical significance of *p* = 0.02 and ** denotes a significance of *p* = 0.04.

Analysis of the recordings detected a significant difference in all frequency bands (delta *p* = 0.03, theta *p* = 0.01, alpha *p* = 0.04, and beta *p* < 0.01) when examining average power per channel per frequency band in the 2 attitude states (calm vs. active) (Table 2). Average power per channel per frequency band was lower when dogs were calm than when they were active. A significant treatment effect (pre- vs. post- stimulation) in average power per channel was observed only in the theta (*p* = 0.02) and alpha (*p* = 0.04) bands (Table 2 and Figure 6), with the average power of both frequency bands decreasing post-stimulation. In the mean frequency analysis, attitude had a significant effect only for the beta and theta bands (*p* ≤ 0.01 for both values). Stimulation had no significant effect on the mean frequency in any band (Supplementary Table 1). No significant interaction was observed between dog, attitude, and stimulation in the multivariate model, neither within the same dog nor between individuals.

**Table 2:** Average power per channel per frequency band with upper and lower confidence intervals for the pre- and post-stimulation periods and for the calm vs active states.

**Frequency band**	**Average power (μV)**	**Lower 95% CI**	**Upper 95% CI**	***p*** **-Value pre vs. post stimulation**	***p*** **-Value calm vs. active**
Delta pre-stimulation	3.31	2.45	4.47	0.23	
Delta post-stimulation	2.9	2.15	3.91		
Delta calm	2.72	2.03	3.65		0.03*
Delta active	3.53	2.59	4.80		
Theta pre-stimulation	1.28	0.95	1.73	0.02*	
Theta post-stimulation	1.05	0.78	1.42		
Theta calm	1.04	0.77	1.40		0.01*
Theta active	1.29	0.95	1.75		
Alpha pre-stimulation	0.88	0.67	1.16	0.04*	
Alpha post-stimulation	0.74	0.57	0.98		
Alpha calm	0.74	0.57	0.97		0.04*
Alpha active	0.88	0.67	1.16		
Beta pre-stimulation	0.85	0.62	1.17	0.61	
Beta post-stimulation	0.78	0.57	1.08		
Beta calm	0.60	0.44	0.82		<0.01*
Beta active	1.10	0.79	1.55		

*CI, confidence interval*.

An * denotes statistical significance.

### EEG Pooled Power Spectral Analysis

**Figure 7:**
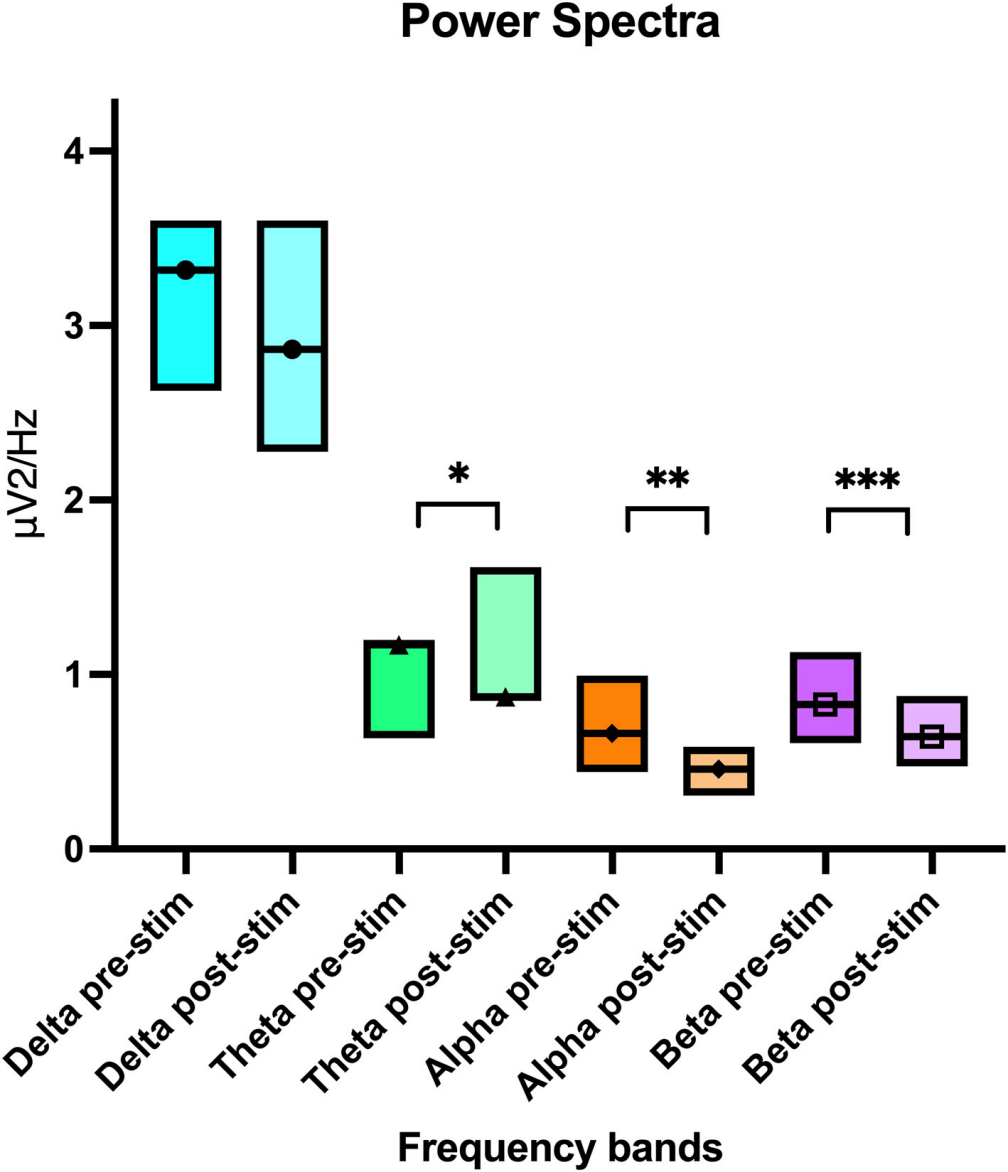
Boxplot graph showing the pooled power spectral analysis mean value per electroencephalogram frequency band with the upper and lower confidence intervals. The line crossing the boxes indicate the median value obtained in that frequency band. An * denotes statistical significance of *p* = 0.01, ** significance of *p* < 0.01 and *** significance of *p* = 0.03.

In this parallel analysis of pooled data, attitude also had a statistically significant effect on all frequency bands (delta *p* ≤ 0.01, theta *p* = 0.01, and beta *p* = 0.01) except for alpha (*p* = 0.91). Similar to the per channel analysis, the power was lower when “calm” vs. when “active” (Table 3). When comparing pre- and post- stimulation mean power values, theta (*p* = 0.01), alpha (*p* ≤ 0.01), and beta (*p* = 0.03) frequencies showed a significant decrease post-stimulation (Table 3 and Figure 7). No significant difference was found in the F50 pre- and post-stimulation (*p* = 0.21) and active vs. calm (*p* = 0.18) nor the F95 results pre- and post-stimulation (*p* = 0.2) and active vs. calm (*p* = 0.84). Dog, attitude, and treatment did not have any significant interaction in the multivariate model. The mean, *p*-values and confidence intervals for all the quantitative analysis have been summarized in Table 3 and Figure 7.

**Table 3:** Pooled power spectral analysis per frequency band with upper and lower confidence intervals for the pre- and post-stimulation periods and for the calm vs. active states.

**Frequency band**	**Mean value (μV^**2**^/Hz)**	**Lower 95% CI values**	**Upper 95% CI values**	***p*** **-Value pre vs. post stimulation**	***p*** **-Value calm vs. active**
Delta pre-stimulation	3.32	2.63	4.2	0.16	
Delta post-stimulation	2.86	2.28	3.60		
Delta calm	2.5	2.01	3.10		<0.01*
Delta active	3.8	2.96	4.88		
Theta pre-stimulation	1.17	0.63	1.2	0.01*	
Theta post-stimulation	0.87	0.85	1.62		
Theta calm	0.89	0.64	1.2		0.01*
Theta active	1.16	0.83	1.62		
Alpha pre-stimulation	0.66	0.44	0.99	<0.01*	
Alpha post-stimulation	0.46	0.31	0.68		
Alpha calm	0.55	0.37	0.81		0.91
Alpha active	0.55	0.37	0.84		
Beta pre-stimulation	0.83	0.61	1.13	0.03*	
Beta post-stimulation	0.64	0.47	0.88		
Beta calm	0.63	0.47	0.85		0.01*
Beta active	0.85	0.61	1.17		
F50 pre-stimulation	4.69	2.84	7.72	0.21	
F50 post-stimulation	3.28	2.01	5.35		
F50 calm	4.75	3.01	7.49		0.18
F50 active	3.23	1.89	5.54		
F95 pre-stimulation	11.55	9.55	13.55	0.20	
F95 post-stimulation	9.97	8.04	11.9		
F95 calm	10.64	8.92	12.35		0.84
F95 active	10.88	8.67	13.1		

*CI, confidence interval*.

An * denotes statistical significance.

### Holter Recordings

**Figure 8:**
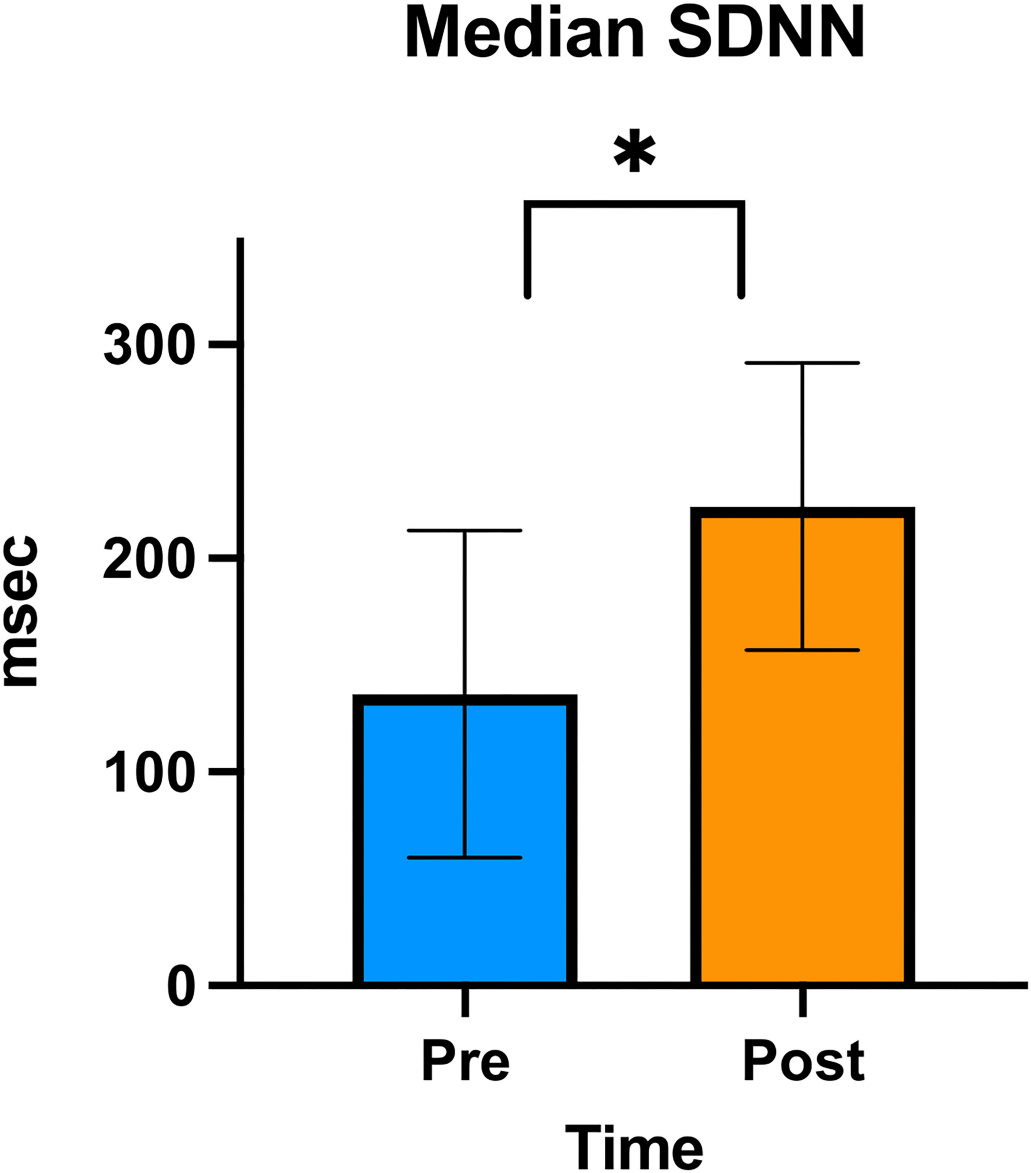
SDNN graph. Column graphic of the SDNN index showing the difference between pre- and post-stimulation as measured on electrocardiogram in the group of 6 dogs. The upper and lower confidence intervals are represented in the graphic. An * denotes statistical significance (*p* < 0.01).

**Figure 9:**
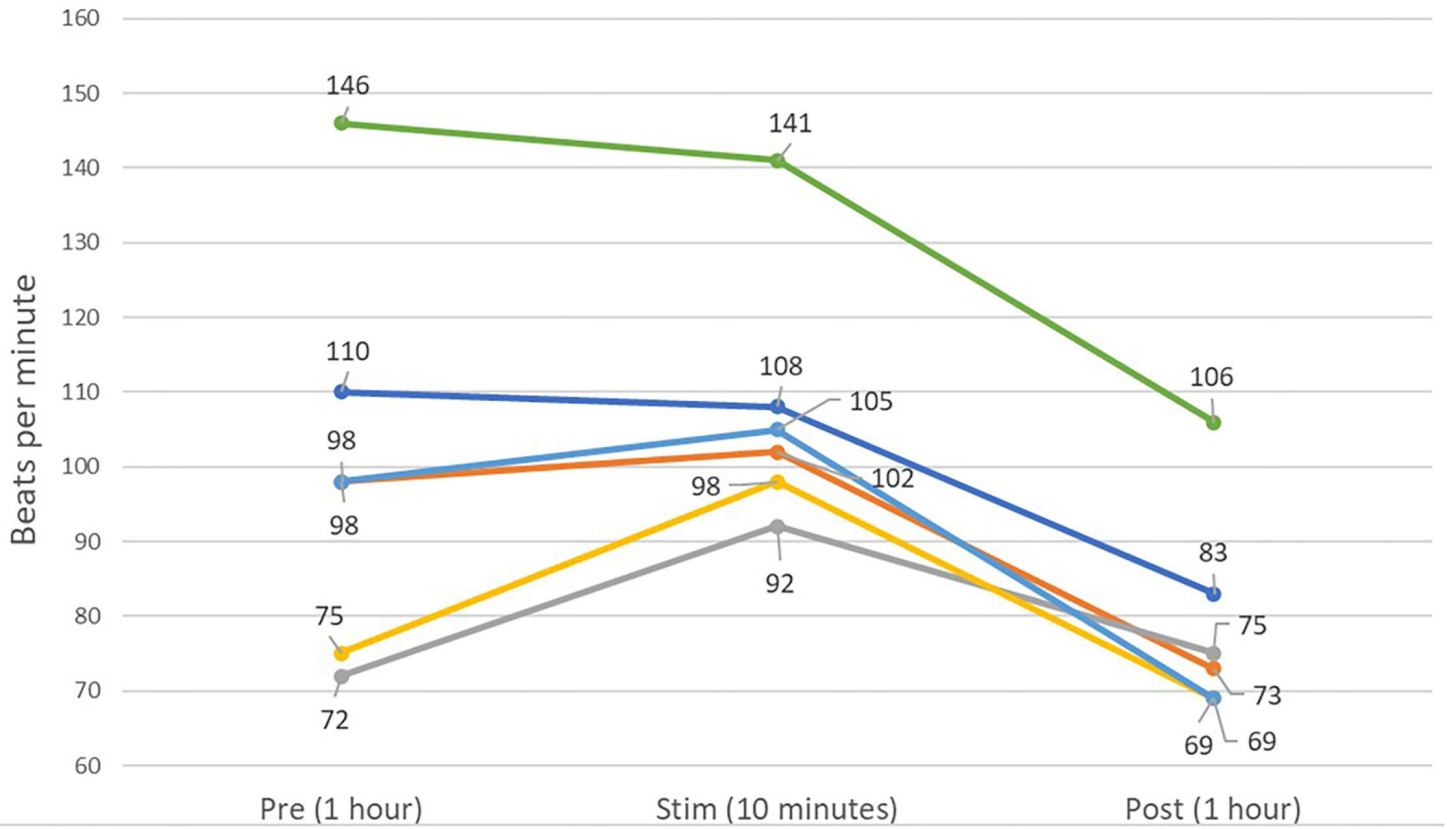
Line graph showing the averaged mean heart rates of the 6 dogs over 1 h of pre-stimulation (Pre) and post-stimulation (Post), as well as the averaged mean heart rate of the 10 min of the peri-stimulation period. A decreased mean heart rate is present in the post-stimulation period for five of six dogs.

**Figure 10:**
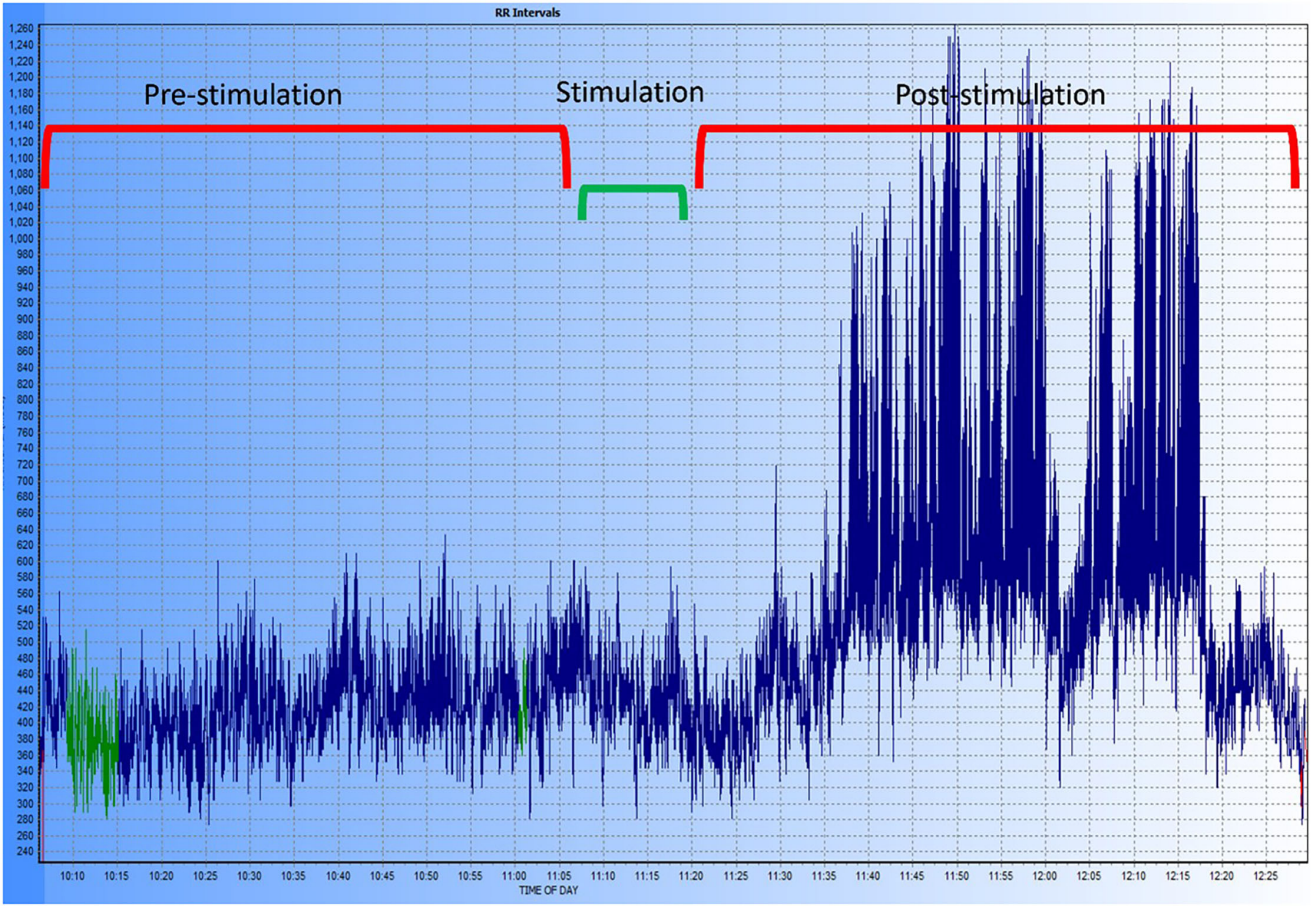
Exemplary tachogram representing the R-R intervals in one of the dogs of the study. The different time periods are shown at the top of the graphic, the period labeled “stimulation” includes the 5-min pre- and post-stimulation margin used for the HRV analysis. A marked increase in HRV can be observed after stimulation and during the entire post-stimulation period recorded.

In the HRV analysis, the SDNN index increased in the post-stimulation period compared to the pre-stimulation period and this difference was statistically significant (pre-stimulation median: 117.26 ms, 95% CI: 71.76–191.61 ms and post-stimulation median 213.96 ms, 95% CI: 130.94–349.61 ms; *p* = 0.01; Figure 8). A significant decrease in the mean heart rate after stimulation was also identified (*p* = 0.01, Table 4 and Figure 9). A tachogram from 1 of the dogs as an exemplary representation of the changes in the SDNN index is shown in Figure 10. The comparison of the mean heart rate and the maximum heart rate of the different 5-min segments (“A”, “B”, “a”, and “b”; Figure 4, Supplementary Table 2) did not reveal any significant differences in the mean and maximum heart rate over time (*p* = 0.096 and *p* = 0.258, respectively).

**Table 4:** Pre- and post-stimulation values, along with the lower and upper 95% confidence intervals for the mean heart rate.

	**Mean heart rate (beats per minute)**	**Lower 95% CI**	**Upper 95% CI**	***p*** **-Value**
Pre	99.83	81	118	* <0.01
Post	79	61	98	

*CI, confidence interval.*

An * denotes statistical significance.

### Tolerability

The stimulation was well-tolerated by the dogs. The responses observed when the stimulation intensity was considered to cause discomfort in the dogs were: pulling away from the apparatus (3 dogs), significant muscle twitching in the neck region (1 dog), and vocalization (1 dog). Once these signs were observed, the level of stimulation was immediately decreased by 0.5 grades. No adverse effects were observed during the 90 s stimulation period, during the post-stimulation period, or during the removal of the EEG and Holter. The owners of the dogs were contacted by one of the investigators (GC) 1 day after the session and then again 1 week after, and no adverse effects were reported.

## Discussion

The EEG and Holter monitoring identified electrophysiologic changes in both brain activity and heart rate and HRV subsequent to tcVNS. The EEG detected decreased power after stimulation in the alpha, beta, and theta frequency bands in line with changes reported in people (38, 39). The Holter monitor identified an increase in the HRV post-stimulation as well as a reduction in the heart rate similar to reports in the human medical literature (50–53).

The decrease in the power of the theta and alpha frequency bands via both methods of power analyses in our study are suggestive of adequate tcVNS, similar to reported EEG changes with VNS/tcVNS in people (38, 39, 54, 55). These acute changes in the EEG are thought to be secondary to the desynchronization of brain activity as afferent vagus nerve signals cause a shift from predominantly low frequency activity (like delta, theta and alpha) to high frequency bands (like beta and gamma) thus promoting a higher arousal/awareness state (38, 39, 54). This shift in the mentation is considered important as seizure activity occurs with synchronized cortical activity, and predominance of slow-waves in delta frequency bands have been observed in interictal and ictal periods (56–58).

The power decrease in the beta frequency band post stimulation has not been reported in people and is in contrast to the mechanism proposed above. It is possible that this finding is a common change observed in the EEG of dogs after tcVNS, although another possibility is habituation of the dog to the surroundings and decreased exploration (“active”) behavior during the second part of the recording, however, this last explanation is considered less likely. Firstly, the epochs for analysis were carefully selected and paired for mental status pre- and post-stimulation; moreover, the multivariate analysis did not find any significant effect of attitude on the power pre- vs. post-stimulation. Additionally, if the decrease in the power of the theta and beta bands was secondary to a more “calmed” mentation, an increase in the delta frequency should also be expected and was not found. A final possibility for the results of this study is that the small sample size of this study skews these findings, thus confirmation with a larger cohort of dogs and a placebo-control group for comparison is required.

In both dogs and people, the resting EEG shows a different prevalence of the various frequency bands depending on the level of alertness of the individual. For instance, a delta or theta rhythm dominates in early sleep or drowsiness while an alpha rhythm predominates in periods of wakefulness when the eyes are closed (36, 41, 59, 60). Ideally, quantitative EEG analysis comparisons are performed on the same mentation state before and after the stimulation, i.e., drowsiness pre- vs. post-stimulation. In dogs, it is not possible to perfectly match this behavior pre- and post-stimulation. Instead, we grouped the dogs' behavior more broadly based on their level of engagement and activity during the analysis of the EEG recording. By pairing epochs with similar mentation states pre- and post-stimulus we tried to reduce as much as possible the influence of the mentation on the analysis of the results. To make the method of assessing mentation as objective as possible, the criteria used for categorizing an epoch as “calm” or “active” were consistent for every dog. Moreover, the multivariate model accounted for “attitude” as a random effect and did not find any significant interaction with the stimulation in the analysis.

One of the measures included in this study as an internal control for the stimulation was the detection of significant differences between “calm” and “active” mentation states. In the average power analysis, there was an increase in the power across all the frequency bands in the “active” epochs selected, regardless of if it was pre- or post-stimulation. The increase in power across the higher frequency bands (beta) is associated with the high level of alertness signified by the engagement of the dogs with their surroundings. A decrease in the low frequency bands might be anticipated in “active” dogs, however the increase power in the low frequency bands in our study could be explained by the fact that most of the physiologic artifacts associated with activity, like eye movements, respiration, perspiration, and whole body movement, present in the low frequency domain of delta and theta (61, 62).

Another internal control measure used in our study was the HRV analysis. Besides the importance of the objective documentation of the effects of tcVNS on the cortex, objective changes were expected in heart rate and HRV due to the vagus nerve's efferent influence on several organs, including the heart. Similar studies in people found that VNS/tcVNS resulted in measurable changes in heart rate and HRV (26, 27, 63, 64). The changes reported in these studies are in line with the results reported in our study. However, a more recent meta-analysis assessing HRV changes with auricular tcVNS found that there is insufficient evidence to support the expectation of a change in this physiologic parameter after auricular stimulation; this stimulation route may not be sufficiently analogous for comparison (65).

Interestingly, a study using a canine heart failure model found that chronic VNS resulted in an increase in HRV assessed by SDNN, as in our study (24). From this perspective, VNS/tcVNS could offer a novel adjunctive treatment modality for those cardiac diseases affecting dogs where parasympathetic dysfunction might contribute to disease presentation or progression, as in heart failure, arrhythmogenic right ventricular cardiomyopathy and dilated cardiomyopathy (24, 31, 32, 66). A previous HRV study in dogs comparing VNS vs. sham stimulation did not find significant difference in HRV between groups (29). It is unclear if the lack of detectable changes in dogs in the aforementioned paper could have been due to the short period of stimulation, the stimulation parameters or if healthy individuals do not show a response to the physiologic effects of vagus nerve stimulation. These findings are contrary to what was found in our study that also used healthy dogs.

To determine whether the decrease in heart rate and increase in HRV were due to habituation to the room over the 2-h study, mean and maximum heart rate were captured from 5-min segments at the beginning and end of each 1-h period (pre- and post-stimulation). Comparisons between these 4 time periods showed no statistically significant difference, suggesting that the HRV changes are more likely to be secondary to the tcVNS itself rather than habituation of the individuals. A decrease in both, mean and maximum heart rate over the 2-h study period would be expected if the dogs progressively became more relaxed.

A previous canine study using tcVNS in epileptic dogs did not find a significant effect on seizure frequency using similar device settings as our study (12). Our findings support that these settings provided adequate stimulation of the vagus nerve to cause detectable physiologic changes. Further, the two studies agree that the apparatus appears to be relatively innocuous and is well tolerated. These findings are encouraging to continue exploring tcVNS and its potential as an effective adjunctive therapy in drug-resistant epilepsy in dogs.

There are important limitations to consider in our study. The study was designed as a pilot study and, because of this novelty, a sample size calculation was not performed. A larger sample size would have allowed to better characterize the normal resting state of the individuals and would have increased or reduced the significant results.

The selection of artifact-free epochs by a blinded investigator as well as their number and length for analysis were established following the recommended guidelines in human studies (41). There are common morphological EEG features between dogs and people, for instance, an 8–12 Hz predominantly alpha rhythm in the occipital area, rostral to caudal (anterior to posterior) gradient of frequencies and similar sleep-associated waveforms (67). Despite these similarities, differences between the 2 species could exist–this needs more exploration. Most of the recent veterinary publications highlight confluences in the epileptogenic waveforms in clinical epileptic syndromes in the 2 species (47, 68–73). Some of the main challenges in veterinary medicine as compared to human epileptology are the lack of consensus on the electrode placement array, number of channels used, and the different skull types. This absence of standardization could lead to incorrect classification of normal EEG patterns into changes or alterations thought to be secondary to the intervention. Future studies replicating or testing our study should ideally try to follow a consistent, validated instrumentation protocol to avoid unnecessary variables.

Currently, information regarding the ideal intensity and duration of stimulation in dogs is missing, therefore, the intensity and duration of stimulation used in this study were those recommended by the manufacturer. Despite a recent study having used the suggested intensity in humans (1.50 mA) to describe the side effects of implantable VNS therapy in dogs, it is still unclear what is the recommended intensity and duration needed for chronic treatment in dogs (74). The design of our pilot study was focused on identifying the acute physiologic changes detectable when using tcVNS, therefore, the comparison of different intensities, durations, and periods of treatments was not performed. Despite this limitation, the intensity and duration of stimulation used in this study resulted in changes in brain activity and HRV.

Finally, due to the design of this study and the number of individuals recruited, the inclusion of a control group was not possible. The inclusion of a control group would have helped to confirm that our results were indeed secondary to the use of the tcVNS and not secondary to other external variables. Future studies should consider the incorporation of a sham-control group to prove the efficacy and/or effects of this therapy.

There are important considerations for future studies considering the use of tcVNS. For instance, the individual's seizure type, client education to ensure adequate delivery of the stimulation, habituating the dog to the tcVNS, establishing the therapeutic or highest tolerable dose for each individual, the number and timing of daily stimulations, and the length of the treatment. The length of the treatment is of particular importance because tcVNS appears to be efficacious after several months of treatment based on studies in people (5, 75, 76). Ideally, future studies looking at tcVNS efficacy in dogs should consider several months of randomized, sham-controlled treatment in epileptics with similar seizure types.

This study revealed that the use of a handheld tcVNS device in healthy dogs could result in measurable and recordable physiologic changes in brain activity and HRV detected by EEG and Holter monitoring, respectively. As a pilot study, the impact of confounding factors was minimized but not completely excluded. These results are encouraging and warrant further investigations to confirm these findings in a larger cohort of dogs and to clarify the potential clinical relevance of this treatment modality.

## Data Availability Statement

The raw data supporting the conclusions of this article will be made available by the authors, without undue reservation.

## Ethics Statement

The study protocol was reviewed and approved by the institutional Animal Care Committee of the University of Guelph (AUP 4265) and follows the Canadian Council on Animal Care Guidelines. Written informed consent was obtained from the owners for the participation of their animals in this study.

## Author Contributions

GC, FJ, and CC: conception and design. GC, LG, SF, GM, and FJ: acquisition of data and drafting the article. GC, LG, SF, CC, GM, and FJ: analysis and interpretation of data, revising article for intellectual content, and final approval of the completed article.

## Funding

Morris Animal Foundation provided funding for the study under the grant number D20CA-840 as well as the Canada Foundation for Innovation and Ontario Ministry of Research, Innovation and Science (#30953). Convergent Medical Technologies, Inc., purchased the gammaCore from electroCore, Inc., and provided it to the investigators for the study. NSERC Discovery Grant Launch Supplement DGECR-2021-00040 funds supported open access publication fees.

## Conflict of Interest

CC is a paid consultant to electroCore, Inc. (Rockaway, NJ, United States) and is co-founder and chief scientist of Convergent Medical Technologies, Inc. (Oyster Bay, NY, United States), which is developing wearable closed-loop neuromodulation therapies for the veterinary market. The remaining authors declare that the research was conducted in the absence of any commercial or financial relationships that could be construed as a potential conflict of interest.

## Publisher's Note

All claims expressed in this article are solely those of the authors and do not necessarily represent those of their affiliated organizations, or those of the publisher, the editors and the reviewers. Any product that may be evaluated in this article, or claim that may be made by its manufacturer, is not guaranteed or endorsed by the publisher.

## Abbreviations

EEG: Electroencephalography
ECG: electrocardiography
HRV: heart rate variability
VNS: vagus nerve stimulation
tcVNS: transcutaneous cervical vagus nerve stimulation
SDNN: standard deviation of the inter-beat N-N interval.

## References

[1] González HFJ Yengo-Kahn A Englot DJ. Vagus nerve stimulation for the treatment of epilepsy. Neurosurg Clin N Am. (2019) 30:219–30. 10.1016/j.nec.2018.12.005 30898273 PMC6432928

[2] Wheless JW Gienapp AJ Ryvlin P. Vagus nerve stimulation (VNS) therapy update. Epilepsy Behav. (2018) 88:2–10. 10.1016/j.yebeh.2018.06.032 30017839

[3] Pérez-Carbonell L Faulkner H Higgins S Koutroumanidis M Leschziner G. Vagus nerve stimulation for drug-resistant epilepsy. Pract Neurol. (2020) 20:189–98. 10.1136/practneurol-2019-002210 31892545

[4] Yuan H Silberstein SD. Vagus nerve and vagus nerve stimulation, a comprehensive review: part II. Headache. (2016) 56:259–66. 10.1111/head.12650 26381725

[5] Liu A Rong P Gong L Song L Wang X Li L. Efficacy and safety of treatment with transcutaneous vagus nerve stimulation in 17 patients with refractory epilepsy evaluated by electroencephalogram, seizure frequency, and quality of life. Med Sci Monit. (2018) 24:8439–48. 10.12659/MSM.910689 30467307 PMC6266629

[6] Redgrave J Day D Leung H Laud PJ Ali A Lindert R. Safety and tolerability of transcutaneous vagus nerve stimulation in humans; a systematic review. Brain Stimul. (2018) 11:1225–38. 10.1016/j.brs.2018.08.010 30217648

[7] Silberstein SD Mechtler LL Kudrow DB Calhoun AH McClure C Saper JR. Non–invasive vagus nerve stimulation for the acute treatment of cluster headache: findings from the randomized, double-blind, sham-controlled ACT1 study. Headache J Head Face Pain. (2016) 56:1317–32. 10.1111/head.12896 27593728 PMC5113831

[8] Yap JYY Keatch C Lambert E Woods W Stoddart PR Kameneva T. Critical review of transcutaneous vagus nerve stimulation: challenges for translation to clinical practice. Front Neurosci. (2020) 14:284. 10.3389/fnins.2020.00284 32410932 PMC7199464

[9] Lane SB Bunch SE. Medical management of recurrent seizures in dogs and cats. J Vet Intern Med. (1990) 4:26–39. 10.1111/j.1939-1676.1990.tb00871.x 2407841

[10] Muñana KR Vitek SM Tarver WB Saito M Skeen TM Sharp NJH. Use of vagal nerve stimulation as a treatment for refractory epilepsy in dogs. J Am Vet Med Assoc. (2002) 221:977–83. 10.2460/javma.2002.221.977 12369700

[11] Martlé V Van Ham LML Boon P Caemaert J Tshamala M Vonck K. Vagus nerve stimulator placement in dogs: surgical implantation technique, complications, long-term follow-up, and practical considerations. Vet Surg. (2016) 45:71–8. 10.1111/vsu.12427 26731597

[12] Robinson K Platt S Stewart G Reno L Barber R Boozer L. Feasibility of non-invasive vagus nerve stimulation (gammaCore VETTM) for the treatment of refractory seizure activity in dogs. Front Vet Sci. (2020) 7:569739. 10.3389/fvets.2020.569739 33195555 PMC7524862

[13] Yuan H Silberstein SD. Vagus nerve and vagus nerve stimulation, a comprehensive review: part I. Headache. (2016) 56:71–8. 10.1111/head.12647 26364692

[14] Uemura E. Medula oblongata. In: Fundamentals of Canine Neuroanatomy and Neurophysiology. Ames, IA: Wiley-Blackwell (2015). p. 201–36.

[15] Skerritt G. King's Applied Anatomy of the Central Nervous System of Domestic Animals. 2nd ed. Oxford: Wiley Blackwell (2018).

[16] Ruffoli R Giorgi FS Pizzanelli C Murri L Paparelli A Fornai F. The chemical neuroanatomy of vagus nerve stimulation. J Chem Neuroanat. (2011) 42:288–96. 10.1016/j.jchemneu.2010.12.002 21167932

[17] Krahl SE Clark KB. Vagus nerve stimulation for epilepsy: a review of central mechanisms. Surg Neurol Int. (2012) 3(Suppl. 4). 10.4103/2152-7806.103015 23230530 PMC3514919

[18] Martlé V Raedt R Waelbers T Smolders I Vonck K Boon P. The effect of vagus nerve stimulation on CSF monoamines and the PTZ seizure threshold in dogs. Brain Stimul. (2015) 8:1–6. 10.1016/j.brs.2014.07.032 25442153

[19] Thayer JF Hansen AL Saus-Rose E Johnsen BH. Heart rate variability, prefrontal neural function, and cognitive performance: the neurovisceral integration perspective on self-regulation, adaptation, and health. Ann Behav Med. (2009) 37:141–53. 10.1007/s12160-009-9101-z 19424767

[20] Yuan H Silberstein SD. Vagus nerve and vagus nerve stimulation, a comprehensive review: part III. Headache. (2016) 56:479–90. 10.1111/head.12649 26364805

[21] Martlé V Peremans K Raedt R Vermeire S Vonck K Boon P. Regional brain perfusion changes during standard and microburst vagus nerve stimulation in dogs. Epilepsy Res. (2014) 108:616–22. 10.1016/j.eplepsyres.2014.02.004 24630046

[22] Krahl SE. Vagus nerve stimulation for epilepsy: a review of the peripheral mechanisms. Surg Neurol Int. (2012) 3:47. 10.4103/2152-7806.91610 22826811 PMC3400480

[23] Randall WC Ardell JL Becker DM. Differential responses accompanying sequential stimulation and ablation of vagal branches to dog heart. Am J Physiol Hear Circ Physiol. (1985) 249:H133–40. 10.1152/ajpheart.1985.249.1.H133 4014479

[24] Zhang Y Popović ZB Bibevski S Fakhry I Sica DA Van Wagoner DR. Chronic vagus nerve stimulation improves autonomic control and attenuates systemic inflammation and heart failure progression in a canine high-rate pacing model. Circ Hear Fail. (2009) 2:692–9. 10.1161/CIRCHEARTFAILURE.109.873968 19919995

[25] Zhang Y Popović ZB Kusunose K Mazgalev TN. Therapeutic effects of selective atrioventricular node vagal stimulation in atrial fibrillation and heart failure. J Cardiovasc Electrophysiol. (2013) 24:86–91. 10.1111/j.1540-8167.2012.02405.x 22913453

[26] De Couck M Cserjesi R Caers R Zijlstra WP Widjaja D Wolf N. Effects of short and prolonged transcutaneous vagus nerve stimulation on heart rate variability in healthy subjects. Auton Neurosci Basic Clin. (2017) 203:88–96. 10.1016/j.autneu.2016.11.003 28017263

[27] Levin C Wai J Perricone A Martinez D. The effect of bilateral transcutaneous vagus nerve stimulation on heart rate variability and impulsivity. Brain Stimul. (2019) 12:523. 10.1016/j.brs.2018.12.719

[28] Borges U Laborde S Raab M. Influence of transcutaneous vagus nerve stimulation on cardiac vagal activity: Not different from sham stimulation and no effect of stimulation intensity. PLoS ONE. (2019) 14:e0223848. 10.1371/journal.pone.0223848 31603939 PMC6788680

[29] Martlé V Bavegems V Van Ham L Boon P Vonck K Raedt R. Evaluation of heart rate variability in dogs during standard and microburst vagus nerve stimulation: a pilot study. Vet J. (2014) 202:651–3. 10.1016/j.tvjl.2014.09.009 25296848

[30] Levy MN. Autonomic interactions in cardiac control. Ann N Y Acad Sci. (1990) 601:209–21. 10.1111/j.1749-6632.1990.tb37302.x 2221687

[31] Spier AW Meurs KM. Assessment of heart rate variability in Boxers with arrhythmogenic right ventricular cardiomyopathy. J Am Vet Med Assoc. (2004) 224:534–7. 10.2460/javma.2004.224.534 14989545

[32] Pereira YM Woolley R Culshaw G French A Martin M. The vasovagal tonus index as a prognostic indicator in dogs with dilated cardiomyopathy. J Small Anim Pract. (2008) 49:587–92. 10.1111/j.1748-5827.2008.00654.x 19006490

[33] von Borell E Langbein J Després G Hansen S Leterrier C Marchant-Forde J. Heart rate variability as a measure of autonomic regulation of cardiac activity for assessing stress and welfare in farm animals - a review. Physiol Behav. (2007) 92:293–316. 10.1016/j.physbeh.2007.01.007 17320122

[34] Shaffer F Ginsberg JP. An overview of heart rate variability metrics and norms. Front Public Heal. (2017) 5:258. 10.3389/fpubh.2017.00258 29034226 PMC5624990

[35] Task force of the european society of cardiology and the North America society of pacing and electrophysiology. Guidelines heart rate variability Standards of measurement, physiological interpretation, and clinical use. Eur Heart J. (1996) 17:354–81. 8737210

[36] Louis EKS, Frey, LC. Electroencephaolgraphy - An Introductory Text. (2016). Available online at: http://www.ncbi.nlm.nih.gov/pubmed/27748095 (accessed November 1, 2019).

[37] Reif PS Strzelczyk A Rosenow F. The history of invasive EEG evaluation in epilepsy patients. Seizure. (2016) 41:191–5. 10.1016/j.seizure.2016.04.006 27131772

[38] Lewine JD Paulson K Bangera N Simon BJ. Exploration of the impact of brief noninvasive vagal nerve stimulation on EEG and event-related potentials. Neuromodulation. (2019) 22:564–72. 10.1111/ner.12864 30288866

[39] Sharon O Fahoum F Nir Y. Transcutaneous vagus nerve stimulation in humans induces pupil dilation and attenuates alpha oscillations. J Neurosci. (2021) 41:320–30. 10.1523/JNEUROSCI.1361-20.2020 33214317 PMC7810665

[40] Kropotov JD. Quantitative EEG, Event-Related Potentials Neurotherapy. 1st edn. Academic Press Inc (2009). Available online at: https://books.google.ca/books?hl=en&lr=&id=szECZPz7FvcC&oi=fnd&pg=PP1&ots=Cp8c0l7NyN&sig=cLpQ4uANRRiR-RpS_9GTgAV4Wjk&redir_esc=y#v=onepage&q&f=false (accessed June 9, 2021).

[41] Babiloni C Barry RJ Bas ßar DE Blinowska KJ Cichocki A Drinkenburg WHIM. International federation of clinical neurophysiology (IFCN)-EEG research workgroup: recommendations on frequency and topographic analysis of resting state EEG rhythms. Part 1: applications in clinical research studies. Clin Neurophysiol. (2020) 131:285–307. 10.1016/j.clinph.2019.06.234 31501011

[42] Saby JN Marshall PJ. The utility of EEG band power analysis in the study of infancy and early childhood. Dev Neuropsychol. (2012) 37:253–73. 10.1080/87565641.2011.614663 22545661 PMC3347767

[43] Schomer DL Lopes da Silva FH. Niedermeyer's Electroencephalography : Basic Principles, Clinical Applications, and Related Fields. 7th edn New York, NY: Oxford University Press (2018).

[44] Dressler O Schneider G Stockmanns G Kochs EF. Awareness and the EEG power spectrum: analysis of frequencies. Br J Anaesth. (2004) 93:806–9. 10.1093/bja/aeh270 15377585

[45] Zhao W Van Someren JW Li C Chen X Gui W Tian Y. EEG spectral analysis in insomnia disorder: a systematic review and meta-analysis. Sleep Med Rev. (2021) 59:101457. 10.1016/j.smrv.2021.101457 33607464

[46] Goadsby PJ de Coo IF Silver N Tyagi A Ahmed F Gaul C. Non-invasive vagus nerve stimulation for the acute treatment of episodic and chronic cluster headache: a randomized, double-blind, sham-controlled ACT2 study. Cephalalgia. (2018) 38:959–69. 10.1177/0333102417744362 29231763 PMC5896689

[47] James FMK Cortez MA Monteith G Jokinen TS Sanders S Wielaender F. Diagnostic utility of wireless video-electroencephalography in unsedated dogs. J Vet Intern Med. (2017) 31:1469–76. 10.1111/jvim.14789 28758239 PMC5598905

[48] Luca JJ Hazenfratz M Monteith G Sanchez A Gaitero LF. Electrode scalp impedance differences between electroencephalography machines in healthy dogs. Can J Vet Res. (2021) 85:309–11. 34602736 PMC8451707

[49] Nonis R D'Ostilio K Schoenen J Magis D. Evidence of activation of vagal afferents by non-invasive vagus nerve stimulation: an electrophysiological study in healthy volunteers. Cephalalgia. (2017) 37:1285–93. 10.1177/0333102417717470 28648089 PMC5680905

[50] Bretherton B Atkinson L Murray A Clancy J Deuchars S Deuchars J. Effects of transcutaneous vagus nerve stimulation in individuals aged 55 years or above: potential benefits of daily stimulation. Aging. (2019) 11:4836–57. 10.18632/aging.102074 31358702 PMC6682519

[51] Lamb DG Porges EC Lewis GF Williamson JB. Non-invasive vagal nerve stimulation effects on hyperarousal and autonomic state in patients with posttraumatic stress disorder and history of mild traumatic brain injury: preliminary evidence. Front Med. (2017) 4:124. 10.3389/fmed.2017.00124 28824913 PMC5534856

[52] Tran N Asad Z Elkholey K Scherlag BJ Po SS Stavrakis S. Autonomic neuromodulation acutely ameliorates left ventricular strain in humans. J Cardiovasc Transl Res. (2019) 12:221–30. 10.1007/s12265-018-9853-6 30560316 PMC6579714

[53] Farmer AD Strzelczyk A Finisguerra A Gourine AV Gharabaghi A Hasan A. International consensus based review and recommendations for minimum reporting standards in research on transcutaneous vagus nerve stimulation (Version 2020). Front Hum Neurosci. (2021) 14:71. 10.3389/fnhum.2020.568051 33854421 PMC8040977

[54] Bodin C Aubert S Daquin G Carron R Scavarda D Mcgonigal A. Responders to vagus nerve stimulation (VNS) in refractory epilepsy have reduced interictal cortical synchronicity on scalp EEG. Epilepsy Res. (2015) 113:98–103. 10.1016/j.eplepsyres.2015.03.018 25986196

[55] Ricci L Croce P Lanzone J Boscarino M. Transcutaneous vagus nerve stimulation modulates EEG microstates and delta activity in healthy subjects. Brain Sci. (2020) 10:668. 10.3390/brainsci10100668 32992726 PMC7599782

[56] Bartolomei F Naccache L. The global workspace (GW) theory of consciousness and epilepsy. Behav Neurol. (2011) 24:67–74. 10.1155/2011/127864 21447900 PMC5377983

[57] Nariai H Matsuzaki N Juhász C Nagasawa T Sood S Chugani HT. Ictal high-frequency oscillations at 80–200 Hz coupled with delta phase in epileptic spasms. Epilepsia. (2011) 52:e130–4. 10.1111/j.1528-1167.2011.03263.x 21972918 PMC3674760

[58] Chou P Wang GH Hsueh SW Yang YC Kuo CC. Delta-frequency augmentation and synchronization in seizure discharges and telencephalic transmission. iScience. (2020) 23:101666. 10.1016/j.isci.2020.101666 33134896 PMC7586134

[59] Bálint A Eleod H Körmendi J Bódizs R Reicher V Gácsi M. Potential physiological parameters to indicate inner states in dogs: the analysis of ECG, and respiratory signal during different sleep phases. Front Behav Neurosci. (2019) 13:207. 10.3389/fnbeh.2019.00207 31607871 PMC6755330

[60] Kis A Szakadát S Kovács E Gácsi M Simor P Gombos F. Development of a non-invasive polysomnography technique for dogs (Canis familiaris). Physiol Behav. (2014) 130:149–56. 10.1016/j.physbeh.2014.04.004 24726397

[61] Urigüen JA Garcia-Zapirain B. EEG artifact removal - State-of-the-art and guidelines. J Neural Eng. (2015) 12:031001. 10.1088/1741-2560/12/3/031001 25834104

[62] Islam MK Rastegarnia A Yang Z. Les méthodes de détection et de rejet d'artefact de l'EEG de scalp : revue de littérature. Neurophysiol Clin. (2016) 46:287–305. 27751622 10.1016/j.neucli.2016.07.002

[63] Mulders DM De Vos CC Vosman I Van Putten MJAM. The effect of vagus nerve stimulation on cardiorespiratory parameters during rest and exercise. Seizure. (2015) 33:24–8. 10.1016/j.seizure.2015.10.004 26524500

[64] Butt MF Albusoda A Farmer AD Aziz Q. The anatomical basis for transcutaneous auricular vagus nerve stimulation. J Anat. (2020) 236:588–611. 10.1111/joa.13122 31742681 PMC7083568

[65] Wolf V Kühnel A Teckentrup V Koenig J Kroemer NB. Does transcutaneous auricular vagus nerve stimulation affect vagally mediated heart rate variability? A living and interactive Bayesian meta-analysis. Psychophysiology. (2021) 58:e13933. 10.1111/psyp.13933 34473846

[66] Oliveira MS Muzzi RAL Araújo RB Muzzi LAL Ferreira DF Nogueira R. Heart rate variability parameters of myxomatous mitral valve disease in dogs with and without heart failure obtained using 24-hour Holter electrocardiography. Vet Rec. (2012) 170:622. 10.1136/vr.100202 22645158

[67] Davis KA Sturges BK Vite CH Ruedebusch V Worrell G Gardner AB. A novel implanted device to wirelessly record and analyze continuous intracranial canine EEG. Epilepsy Res. (2011) 96:116–22. 10.1016/j.eplepsyres.2011.05.011 21676591 PMC3175300

[68] Jaggy A Bernardini M. Idiopathic epilepsy in 125 dogs: a long-term study. Clinical and electroencephalographic findings. J Small Anim Pract. (1998) 39:23–9. 10.1111/j.1748-5827.1998.tb03665.x 9494931

[69] Holliday TA Williams DC. Interictal paroxysmal discharges in the electroencephalograms of epileptic dogs. Clin Tech Small Anim Pract. (1998) 13:132–43. 10.1016/S1096-2867(98)80034-0 9775503

[70] Berendt M Høgenhaven H Flagstad A Dam M. Electroencephalography in dogs with epilepsy: similarities between human and canine findings. Acta Neurol Scand. (1999) 99:276–83. 10.1111/j.1600-0404.1999.tb00676.x 10348156

[71] Wielaender F James FMK Cortez MA Kluger G Neßler JN Tipold A. Absence seizures as a feature of juvenile myoclonic epilepsy in rhodesian ridgeback dogs. J Vet Intern Med. (2018) 32:428–32. 10.1111/jvim.14892 29194766 PMC5787207

[72] Poma R Ochi A Cortez MA. Absence seizures with myoclonic features in a juvenile Chihuahua dog. Epileptic Disord. (2010) 12:138–41. 10.1684/epd.2010.0312 20483714

[73] Morita T Shimada A Takeuchi T Hikasa Y Sawada M Ohiwa S. Cliniconeuropathologic findings of familial frontal lobe epilepsy in Shetland sheepdogs. Can J Vet Res. (2002) 66:35–41. 11858647 PMC226980

[74] Harcourt-Brown TR Carter M. Implantable vagus nerve stimulator settings and short-term adverse effects in epileptic dogs. J Vet Intern Med. (2021) 35:2350–8. 10.1111/jvim.16226 34472639 PMC8478022

[75] Bauer S Baier H Baumgartner C Bohlmann K Fauser S Graf W. Transcutaneous vagus nerve stimulation (tVNS) for treatment of drug-resistant epilepsy: a randomized, double-blind clinical trial (cMPsE02). Brain Stimul. (2016) 9:356–63. 10.1016/j.brs.2015.11.003 27033012

[76] Barbella G Cocco I Freri E Marotta G Visani E Franceschetti S. Transcutaneous vagal nerve stimulation (t-VNS): an adjunctive treatment option for refractory epilepsy. Seizure. (2018) 60:115–9. 10.1016/j.seizure.2018.06.016 29940349

