# Supplementary material for: Transcutaneous Cervical Vagus Nerve Stimulation Induces Changes in the Electroencephalogram and Heart Rate Variability of Healthy Dogs, a Pilot Study
Source: Front Vet Sci. 2022 Jun 13;9:878962. doi: 10.3389/fvets.2022.878962 (PMC9234651; doi:10.3389/fvets.2022.878962)
Supplement: Supplementary file 1 [file Data_Sheet_1.PDF]

Supplementary table 1

Mean frequency values per channel per frequency band with the upper and lower confidence intervals for the pre- and post-stimulation periods and for the calm vs active behavior. An * denotes statistical significance.

Frequency band	Mean frequency (Hz)	Upper 95% CI	Upper 95% CI	p value pre vs post stimulation	p value calm vs active
Delta pre-stimulation	2.08	2.0	2.17	0.28	
Delta post-stimulation	2.03	1.95	2.12		
Delta calm	2.03	1.95	2.11		0.17
Delta active	2.09	2	2.18		
Theta pre-stimulation	5.64	5.58	5.71	0.91	
Theta post-stimulation	5.64	5.57	5.70		
Theta calm	5.72	5.66	5.78		<0.01*
Theta active	5.56	5.48	5.63		
Alpha pre-stimulation	10.36	10.14	10.58	0.74	
Alpha post-stimulation	10.38	10.16	10.60		
Alpha calm	10.30	10.09	10.52		0.06
Alpha active	10.43	10.21	10.66		
Beta pre-stimulation	21.22	10.73	21.71	0.37	
Beta post-stimulation	21.38	20.90	21.88		
Beta calm	20.88	20.42	21.35		<0.01*
Beta active	21.73	21.22	22.25		

CI: confidence interval

Supplementary table 2

Maximum and mean heart rate values for the different 5-minute segments. The different 5 minute-segments are labeled “A”, “B”, “a” and “b” according to Figure 4.

Dog	Time	Maximum HR (bpm)	Mean HR (bpm)
1	A	109	106
2	A	152	135
3	A	121	110
4	A	86	82
5	A	88	82
6	A	162	159
1	B	142	126
2	B	88	84
3	B	44	41
4	B	94	66
5	B	86	82
6	B	132	129
1	a	74	66
2	a	88	79
3	a	86	75
4	a	62	58
5	a	56	51
6	a	136	95
1	b	73	71
2	b	103	87
3	b	80	71
4	b	85	76
5	b	100	97
6	b	158	154

HR: heart rate, bpm: beat per minute
